# Partial response to first generation SSA guides the choice and predict the outcome of second line therapy in acromegaly

**DOI:** 10.1007/s12020-022-03158-w

**Published:** 2022-08-20

**Authors:** Sabrina Chiloiro, Denise Costa, Rosa Lauretta, Valeria Mercuri, Emilia Sbardella, Irene Samperi, Marialuisa Appetecchia, Antonio Bianchi, Antonella Giampietro, Patrizia Gargiulo, Andrea M. Isidori, Maurizio Poggi, Alfredo Pontecorvi, Laura De Marinis

**Affiliations:** 1 Department of Translational Medicine and Surgery, Università Cattolica del Sacro Cuore, 00168 Roma, Italy; 2 UOC Endocrinology and Diabetology, Fondazione Policlinico Universitario A. Gemelli IRCCS, 00168 Roma, Italy; 3 Department of Experimental Medicine, Endocrinology-Pituitary Disease, “Sapienza” University of Rome, Roma, Italy; 4 Oncological Endocrinology Unit, IRCCS Regina Elena National Cancer Institute, Roma, Italy; 5 Endocrine-Metabolic Unit, Sant’Andrea University Hospital, Rome, Italy

**Keywords:** GH secreting pituitary adenoma, GH, IGF-I, Growth hormone, Pegvisomant, Pasireotide

## Abstract

**Introduction:**

Treatment of acromegaly resistant to first generation somatostatin analogues (first gen-SSA) is often difficult. We aimed to investigate the role of partial response and resistance to first gen-SSA in the choice of second line treatments and their outcomes.

**Patients and methods:**

A retrospective and multicenter study was conducted on 100 SSA-resistant acromegaly patients and treated with Pasireotide Lar (Pasi-Lar), Peg-V in monotherapy (m-Peg-V) or in combination with first gen-SSA (c-Peg-V).

**Results:**

Thirty-three patients (33%) were treated with m-Peg-V, 36 (36%) with c-Peg-V and 31 with Pasi-Lar (31%). According to logistic regression, m-Peg-V was chosen in older patients (*p* = 0.01) and with not-invasive adenomas (*p* = 0.009), c-Peg-V therapy in younger patients (*p* = 0.001), with invasive adenomas (*p* = 0.02), Pasi-Lar was in invasive adenomas (*p* = 0.01) and in patients partially responsive to first-gen SSA (*p* = 0.01). At the last follow-up, 68 patients (68%) reached the acromegaly control: 22 with m-Peg-V (32.4%), 23 with c-Peg-V (33.8%) and 23 with Pasi-Lar (33.8%). Patients non-responsive to c-Peg-V had higher IGF-I levels (median 3.2 x ULN, IQR: 1.6, *p* < 0.001) and required higher Peg-V dosage (median 30 mg/daily IQR: 10, *p* = 0.002) as compared to responsive patients (median IGF-I x ULN: 2.1 IQR: 1.4; median Peg-V dosage 20 mg/daily IQR: 10). All patients responsive to Pasi-Lar were partially responsive to first gen-SSAs (*p* = 0.02).

**Conclusion:**

Our data showed that c-Peg-V and Pasi-Lar are chosen for the treatment of invasive tumors. The partial response to first gen-SSA seems to be the main determinant for the choice of Pasi-Lar and positively predicts the treatment outcome.

## Introduction

Acromegaly is a chronic, systemic and complex disease, with an increased risk of mortality due to the disease per-se and to the increased prevalence of systemic complications [1]. The reaching of the biochemical control of the growth hormone (GH) and insulin like growth factor-I (IGF-I) hyper-secretion and the management of the tumor mass are the main aims in the treatment of acromegaly, together with the control of disease complication and the improvement of quality of life [2].

The surgical removal of the pituitary adenoma and the medical treatments with first generation somatostatin analogues (first gen-SSAs) represent the cornerstones in the management of acromegaly. According to the most recent meta-analysis, first gen-SSAs can induce the control of hormonal hypersecretion from 40% to 65% of acromegaly patients [3]. As for the consequences, a not negligible group of patients is considered partially or completely resistant to the treatment with first gen-SSA [4]. Partial resistance to first gen-SSAs is defined in cases who did not reach the normalization of GH and IGF-I but at least with reached a significant decrease (>50%) and/or in cases of tumor shrinkage >20% [4]. Complete resistance is instead defined in cases of the absence of normalization of GH and IGF-I levels with a reduction of < 50% with respect of pre-treatment values and without tumor shrinkage [4]. Patients resistant to first generation SSA required treatment with second-line and multi-modal therapies, such as GH receptor antagonist (Pegvisomant) and second generation SSAs (Pasireotide long-acting release). In the absence of conclusive guidelines on the treatments of acromegaly patients, guidelines and consensus were published by the scientific societies, suggesting the orientation of the second line treatment according to the patient’s clinical conditions, biology and morphology of the GH secreting pituitary adenomas and to the patient’s comorbidities [2, 5–7]. So, a retrospective, observational and real-life study was designed to describe the use of second line therapies (Pegvisomant in monotherapy or in combination with SSA and Pasireotide Lar) in four Italian hospitals in terms of patients’ characterizations, long-term outcomes, adverse event rates and physicians’ choice of second line therapies.

## Material and methods

An epidemiological, retrospective, and multicentre study was conducted on clinical data of acromegaly patients followed-up at four hospital-based endocrinology centres in Rome, Italy. Data were retrospectively collected. Data were anonymized, recorded on electronic forms by physicians involved in the patients’ care for each endocrine centre and sent to the Coordinating Centre for analysis. Data collection was conducted between November 2018 and November 2019. Patients involved in the study signed an informed consensus.

*Inclusion criteria*

- patients with active acromegaly at the moment of prescription of second line treatments, such as Pegvisomant or Pasireotide Lar;
- partial response or resistance to first generation SSA
- treatment with PEG-V in monotherapy (m-Peg-V) or in combination with first gen-SSAs (c-Peg-V) or treatment with Pasireotide Lar for at least 12 consecutive months.

*Exclusion criteria*

- History of radiotherapy within 10 years before the prescription of Pasireotide LAR or Pegvisomant;
- treatment with Pegvisomant or Pasireotide Lar in clinical trials
- duration of treatment with Pegvisomant or Pasireotide Lar in clinical trials shorter than 12 months.

### Partial response and resistance to first generation SSA

Partial response to first generation SSA was defined in patients who did not achieve the normalization of GH and IGF-I but with a significant decrease of GH and IGF-I levels (at least by 50% in comparison to pre-treatment levels) and/or in patients with a tumor shrinkage of at least 20%. Poor response or resistance to first generation SSA was defined in patients without a significant decrease of GH and IGF-I levels (less than 50% in comparison to pre-treatment levels) and in the absence of tumor shrinkage [4]. Response to SSA therapy was tested after no less than 6 consecutive months of treatment with first generation SSA at the maximum tolerated dose.

### Study design

Baseline was to the visit/day of the first dose of Pegvisomant or Pasireotide LAR. The treatment choice for Pegvisomant or Pasireotide LAR was based on the phsyician’s clinical judgment in the absence of reference guidelines, which were first published in October 2018 [2]. During the treatment, patients were tested for IGF-I, GH levels; and performed pituitary contrasted MRI according to the clinical practice of each center. According to inclusion criteria, treatment with Pegvisomant or Pasireotide Lar had to last no less than 12 months, for this study. The end of the study corresponded to the last evaluation visit, when all patients had hormonal test and pituitary magnetic resonance images (MRI).

### Invasive GH secreting pituitary adenoma

GH secreting pituitary adenomas were considered as invasive in the cases of invasion of cavernous sinus and/or of the III ventricle. Cavernous sinus invasion was considered for tumors that extended laterally to the lateral tangent of the intra- and supra-cavernous internal carotid artery and in cases of total encasement of the intra-cavernous carotid artery (grade 3 and 4 of Knosp classification) [8]. Invasion was diagnosed through pituitary MRI and confirmed intra-operatory.

### Primary objective

The primary objective of the study was to investigate if the partial response and the resistance to first-generation SSA impacted on the choice of second line treatments in acromegaly patients. As secondary objectives, we investigated if other clinical determinants (such as age, gender, GH and IGF-I levels, tumor dimension and invasion and comorbidities) may impact in choosing second line therapies and may predict the response to second line treatments.

### Endpoints

The primary endpoint of this study was the number of patients treated with Pegvisomant or Pasireotide LAR. According to the treatment choice, patients were divided in three groups:

- patients treated with Pegvisomant in monotherapy;
- patients treated with Pegvisomant in association to first generation SSA;
- patients treated with Pasireotide Lar.

The secondary endpoint of this study was the number of patients who reached the control of acromegaly, in the three groups of treatments. At the last follow-up, patients were defined as controlled if the IGF-I values were in the reference ranges for age and gender (at least in two consecutive measures) and random GH was below 1.0 ng/mL [2]. Patients on treatment with Pegvisomant were evaluated only by serum IGF-I. IGF-I was expressed as IGF-I for the upper limit of normality (ULN) [2].

### Statistical analysis

The patients’ cohort was described in its clinical and demographic features using descriptive statistics techniques. Normality of continuous variables was checked using the Kolmogorov-Smirnov test. Quantitative variables were expressed as median and range and qualitative variables as absolute and percentage frequency. Chi square test (or Fisher exact test when necessary) and Mann Whitney non-parametric tests were used to compare categorical and quantitative un-paired data. Bonferroni correction was applied as appropriate. The variables that reached a statistical significance at the univariate analysis entered the logistic regression. The analyses were performed using SPSS software version 24.0 for Windows.

## Results

The study population included 100 patients. Clinical features of the study population are summarized in Table 1. Forty-one were females and 59 were males (respectively 41% and 59%). Median age at acromegaly diagnosis was 38 (IQR: 19.5). Median GH value and IGF-I x ULN at acromegaly diagnosis were respectively 5 (IQR: 4.3) and 3.4 x ULN (IQR: 1.4). Eighty-two patients (82%) carried a macroadenomas. Forty-eight patients (48%) carried an invasive neoplasia. Seventy-nine patients (79%) underwent pituitary surgery: 71 patients were naïve to medical treatment and the remaining 8 patients had undergone pre-treatment with first gen-SSA. Seventy-eight patients (78%) carried a macroscopic residual of the adenoma. All patients were treated with first generation SSA. Sixty-seven patients were considered partially responsive to first generation SSA (67%) and the remaining 33 were considered resistant to first generation SSA (33%).

**Table 1:** Demographic, clinical and morphological features of acromegaly patients enrolled into the study and univariate analysis according to the treatment groups

					*p*-value		
	Whole cohort (100 pts)	m-Peg-V (33 pts)	c-Peg-V (36 pts)	Pasi- Lar (31 pts)	m-Peg-V vs c-Peg-V	m-Peg-V vs Pasireotide	c-Peg-V vs Pasireotide
**Age at acromegaly diagnosis, median (IQR)**	38 (19.5)	39.5 (17)	34.5 (17.5)	42 (31)	n.s	n.s.	n.s.
**Gender**							
Females *n*, (%)	41 (41%)	11 (33.3%)	17 (47.2%)	13 (41.9%)	n.s.	n.s.	n.s.
Males *n*, (%)	59 (59%)	22 (66.7%)	19 (52.8%)	18 (58.1%)			
**BMI Kg/m2, median (IQR)**	28 (5)	28 (3)	31.5 (7)	30 (3)	n.s	n.s	n.s
**Median GH at acromegaly diagnosis ng/mL, median (IQR)**	5 (4.3)	4.3 (3.3)	6.4 (5.5)	4.4 (5.5)	0.01	n.s	0.005
**Median IGF-I x ULN at acromegaly diagnosis, median (IQR)**	3.4 (1.4)	3.1 (2.2)	3.7 (1.7)	3 (1.6)	n.s	n.s	n.s
**Tumor dimension**							
Micro-adenoma *n*, (%)	18 (18%)	7 (21.2%)	7 (19.4%)	4 (12.9%)	n.s	n.s	n.s
Macro-adenoma *n*, (%)	82 (82%)	26 (78.8%)	29 (80.6%)	27 (87.1%)			
**Invasive tumour**							
Yes *n*, (%)	48 (48%)	7 (21.2%)	19 (52.8%)	22 (71%)	0.007	< 0.001	n.s.
No *n*, (%)	52 (52%)	26 (78.8%)	17 (47.2%)	9 (29%)			
**Residual disease**							
Yes *n, (%)*	78 (78%)	27 (81.8%)	25 (69.4%)	26 (83.9%)	n.s	n.s	n.s
No *n, (%)*	22 /22%)	6 (18.1%)	11 (30.6%)	5 (16.1%)			
**Length of active disease, months median (IQR)**	19.5 (33)	17.5 (17)	27 (51)	30 (84)	n.s	n.s	n.s
**Age at second line treatment, years median (IQR)**	43.5 (22)	50 (21)	39 (11)	44 (27)	0.002	0.04	n.s.
**GH at second line treatment ng/mL, median (IQR)**	4.4 (10)	2.6 (7)	4.8 (14)	4.2 (5)	0.02	n.s	0.004
**IGF-I x ULN at second line treatment, median (IQR)**	2 (1.3)	1.8 (1.7)	3 (3)	1.4 (0.3)	n.s	n.s	n.s
**Systemic comorbidities**							
Systemic arterial hypertension *n*, (%)	36 (36%)	12 (37.5%)	12 (33.3%)	12 (38.7%)	n.s	n.s	n.s
Cardiomyopathy *n*, (%)	29 (29%)	9 (30%)	13 (36.1%)	7 (22.6%)	n.s	n.s	n.s
Diabetes mellitus *n*, (%)	28 (28%)	7 (25%)	11 (30.5%)	10 (35.4%)	n.s	n.s	n.s
Obstructive sleep apnoea syndrome *n*, (%)	17 (17%)	2 (15%)	13 (36.1%)	2 (9.6%)	0.003	n.s	0.004
Nodular thyroid disease *n*, (%)	42 (42%)	9 (30%)	16 (44.4%)	17 (61.3%)	n.s	0.02	n.s
Osteomalacia/osteoporosis *n*, (%)	19 (19%)	2 (10%)	6 (16.7%)	11 (38.7%)	n.s.	0.003	n.s
Second neoplasia *n*, (%)	13 (13%)	4 (15%)	6 (16.7%)	3 (9.6%)	n.s.	n.s	n.s
**Acromegaly outcome with second line therapies**							
Controlled *n*, (%)	68 (68%)	22 (66.7%)	23 (63.9%)	23 (74.2%)	n.s	n.s	n.s
Active *n*, (%)	32 (32%)	11 (33.3%)	13 (36.1%)	8 (25.8%)			

*n.s.* not stastical significant

Thirty-three patients were treated with Pegvisomant in monotherapy (33%), 36 with Pegvisomant plus first gen-SSAs (36%) and 31 with Pasireotide Lar (31%). The initial median dose of Pegvisomant was 10 mg/daily (IQR: 5 mg/daily) in m-Peg-V group and 15 mg/daily (IQR: 10 mg/daily). Fifteen patients were treated with Pasireotide Lar 40 mg/monthly and the remaining 16 patients were treated with Pasireotide Lar 60 mg/monthly (respectively 48.4% and 51.4%).

Thirty-six patients were affected by systemic arterial hypertension (36%), 29 by cardiomyopathy (29%), 28 patients by diabetes mellitus (28%), 17 patients by obstructive sleep apnoea syndrome (17%), 42 patients by nodular thyroid disease (42%), 19 patients by osteoporosis/osteomalacia (19%) and 13 patients (13%) had an history of second neoplasia (breast cancer in 3 patients, thyroid cancer in 5 patients, lung cancer in 1 patient, prostate cancer in 2 patients and kidney cancer in 2 patients). During the treatments, a transaminase elevation (3 times upper than the limit of normality) was observed in 7 patients (7% of cases: 4 were on treatment with m-Peg-V and 3 on c-Peg-V), an injection-site reaction in 2 patients (2%) and a regrowth of residual tumour in 3 patients (2.8% of cases: one case was observed for each group of treatment).

As showed in Table 1, we found that patients on treatment with c-Peg-V were younger at the moment of the prescription of second line therapy (39 years IQR: 11), as compared to patients treated with m-Peg-V (50 years IQR: 21 *p* = 0.002) and to patients treated with Pasireotide Lar (44 years IQR: 27 *p* = 0.04). Moreover, GH values at acromegaly diagnosis were higher (median 6.4 ng/mL IQR: 5.5) in patients treated with c-Peg-V as compared to those treated with m-Peg-V (median 4.3 ng/mL IQR: 3.3 *p* = 0.01) and to those treated with Pasireotide Lar (median 4.4 ng/mL IQR: 5.5 *p* = 0.005). In parallel, we found that GH values after treatment with first gen-SSA were higher in patients treated with c-Peg-V (median 4.8 ng/mL IQR: 14) as compared to those of patients treated with m-Peg-V (median 2.6 ng/mL IQR: 7, *p* = 0.002).

Patients treated with c-Peg-V or Pasireotide Lar more frequently carried an invasive pituitary adenoma (respectively 52.8% *p* = 0.007 and 71% *p* < 0.001) with respect to patients treated with m-Peg-V (21.2%). Moreover, we found that patients treated with c-Peg-V were more frequently affected by obstructive sleep apnoea syndrome (OSAS) (36.1%), with respect of patients treated with m-Peg-V (15%, *p* = 0.003) and Pasireotide Lar (9.6%, *p* = 0.004). Patients treated with Pasireotide Lar instead were more frequently affected by osteoporosis/osteomalacia (38.7%) and nodular thyroid disease (61.3% *p* = 0.02), with respect to those treated with m-Peg-V (frequency of osteoporosis/osteomalacia: 10% *p* = 0.003; frequency of nodular thyroid disease: 30% *p* = 0.02).

**Fig. 1:**
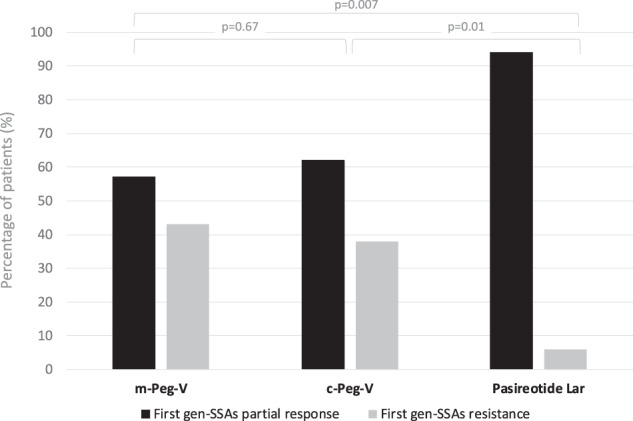
Histogram representing the rate of partial control and resistance of first generation SSA according to the choice of different second line treatments (m-Peg-V, c-Peg-V and Pasireotide Lar). Univariate analysis

In addition, as showed in the Fig. 1, Pasireotide Lar was chosen more frequently for patients considered partial responsive to first gen-SSA (94.1% of cases). At the same time, Peg-V in monotherapy and Peg-V in combination with first gen-SSA were significantly and more frequently prescribed in patients considered resistant to the treatment with first gen-SSA (respectively 43.4% *p* = 0.07 and 37.9% *p* = 0.01).

**Fig. 2:**
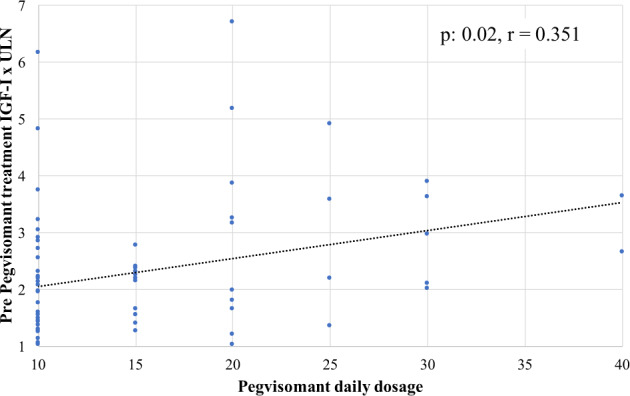
Scatter chart for IGF-I x ULN levels before starting Peg-V and the dosage of Peg-V required to reach the disease control (*p*: 0.02, *r* = 0.351)

At the last follow-up visit, 68 patients reached the acromegaly control (68%), without any significant difference among groups of treatment. We analysed the clinical, hormonal and morphological features to identify potential predictors of second line medical treatments, as showed in Table 2. In particular, we found higher values of IGF-I x ULN at baseline, more frequent large pituitary tumors and more frequent resistance to first generation SSA in patients with active acromegaly during treatment with c-Peg-V as compared to those reported in patients that reached the disease control. In addition, we found a positive correlation between the Peg-V dosage required for reaching the acromegaly control and the levels IGF-I x ULN collected before starting the Peg-V treatment (*p*: 0.02, *r* = 0.351), as shown in Fig. 2.

**Table 2:** Univariate analysis of clinical, hormonal and morphologic features, according to the acromegaly outcome and treatment groups

	m-Peg-V monotherapy			Peg-V plus SSA			Pasireotide Lar		
	Controlled	Active	*p*-value	Controlled	Active	*p*-value	Controlled	Active	*p*-value
**Age at acro-diagnosis, median (IQR**)	32 (13)	41 (20)	0.94	29 (19)	38 (15)	0.38	38.5 (35)	60 (20)	0.723
**Gender**									
Females *n,* (%)	8 (36.4%)	3 (27.3%)	0.486	11 (47.8%)	6 (46.2%)	0.923	8 (34.8%)	5 (62.5%)	0.182
Males *n,* (%)	14 (63.6%)	8 (72.7%)		12 (52.2%)	7 (53.8%)		15 (65.2%)	3 (37.5%)	
**BMI Kg/m2, median (IQR)**	28 (4)	27 (1)	0.98	31 (6)	32 (8)	0.23	30 (4)	29 (1)	0.711
**GH at acro-diagnosis**	6.5 (14)	15 (20)	0.104	6.2 (4.3)	30 (10)	0.37	4 (4)	7.4 (14)	0.381
**ng/mL, median (IQR)**									
**IGF-I x ULN at acro-diagnosis, median (IQR)**	2.2 (1)	3.7 (2)	0.765	3.4 (2)	4.1 (1.5)	0.178	2.4 (1.5)	3 (2.4)	0.368
**Tumor dimension**									
Micro-adenoma *n,* (%)	5 (22.7%)	2 (18.2%)	0.523	7 (30.4%)	0 (0%)	0.03	3 (13%)	1 (2.5%)	0.691
Macro-adenoma *n,* (%)	17 (77.3%)	9 (81.8%)		16 (69.6%)	13 (100%)		20 (87%)	7 (87.5%)	
**Invasive tumour**									
Yes *n*, (%)	4 (18.2%)	3 (27.3%)	0.547	10 (43.5%)	9 (69.2%)	0.137	17 (73.9%)	5 (62.5%)	0.374
No *n,* (%)	18 (81.8%)	8 (72.7%)		13 (56.5%)	4 (30.8%)		6 (26.1%)	3 (37.5%)	
**Residual disease**									
Yes *n,* (%)	17 (77.3%)	10 (91%)	0.338	14 (60.9%)	11 (84.9%)	0.133	19 (82.6%)	7 (87.5%)	0.65
No *n,* (%)	5 (22.7%)	1 (9%)		9 (39.1%)	2 (15.4%)		4 (17.4%)	1 (12.5%)	
**Response to first generation SSA**									
Partial *n*, (%)	13 (59.1%)	6 (54.5%)	0.678	19 (82.6%)	4 (30.7%)	0.07	23 (100%)	5 (62.5%)	0.02
Resistance *n*, (%)	9 (40.9%)	5 (45.5%)		4 (17.4%)	9 (69.3%)		0 (0%)	3 (37.5%)	
**Age at second line treatment**, **years median (IQR)**	53 (20)	43 (9)	0.905	43 (7)	34 (7)	0.179	42.5 (25)	50 (24)	0.848
**GH at second line treatment** **ng/mL, median (IQR)**	1.9 (7)	2.2 (5)	0.481	3.6 (4)	4 (5)	0.131	3.7 (17)	5.2 (7)	0.721
**IGF-I x ULN at second line treatment, median (IQR)**	1.6 (1.6)	2.9 (1)	0.5	2.1 (1.4)	3.2 (1.6)	0.001	1.5 (3)	2 (0.7)	0.339
**Peg-V daily dosage, median (IQR)**	15 (10)	20 (15)	0.57	20 (10)	30 (10)	0.002	Na	Na	Na
**Pasireotide Lar dosage**									
40 mg/monthly *n*, (%)	Na	Na	Na	Na	Na	Na	10 (43.5%)	5 (62.5%)	0.3
60 mg/monthly *n*, (%)							13 (56.5%)	3 (37.5%)	

*n.s.* not stastical significant

**Fig. 3:**
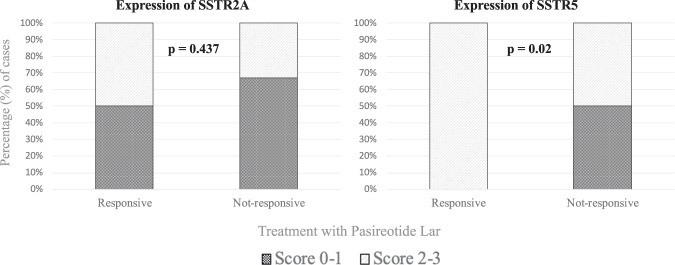
Histogram representing the frequency of expression of the SSTR2A and SSTR5 according to the response to treatment with Pasireotide Lar. Univariate analysis (Fisher test)

According to the multicenter and retrospective design of our study, data on the SSTR expression were available only for 27 cases (9 treated with c-Peg-V and 18 treated with Pasireotide Lar). In the group of the 12 patients responsive to Pasireotide Lar, the expression of the SSTR2A was focal and cytoplasmatic (grade 0 and 1 of Volante score) in 6 cases (50%) and diffuse and membranous (grade 2 and 3 of Volante score) in the remaining 6 cases (50%, *p* = 0.437). Instead, the expression of the SSTR5 diffuse and membranous (grade 2 and 3 of Volante score) in all 12 cases (100%, *p* = 0.02), as shown in Fig. 3.

**Fig. 4:**
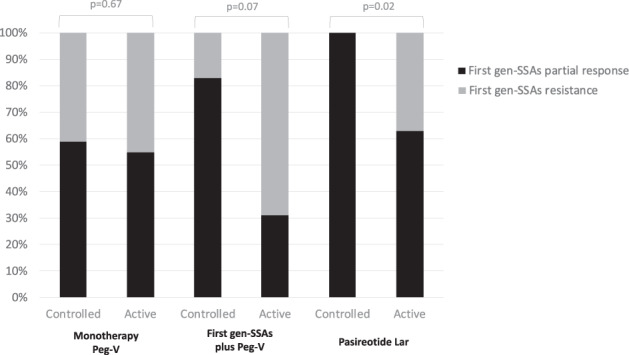
Histogram representing the rate of patients reached the acromegaly control, according to the partial response or resistance of first generation SSA. Univariate analysis

Among the 31 patients treated with Pasireotide Lar, all patients with controlled diseases were considered partially responsive to first-generation SSA, as shown in Fig. 4. The complete resistance to SSA is a risk factor for a poor response to Pasireotide Lar treatment (OR: 1.5, 95% IC: 1.1–3.3; *p* = 0.02).

To better explore the efficacy of Pasireotide Lar in short-term follow-up, we also investigated the percentage of patients who reached the acromegaly control at 6 months of treatment. To reach this secondary objective, we analysed the efficacy of Pasireotide Lar at 6 months of treatment in the present cohort of 31 patients treated with Pasireotide Lar and in a small group of other 10 acromegaly patients treated for at least 6 months, but that did not reach the 12 consecutive months of treatment. As for consequence, a group of 41 acromegaly patients was finally retrospectively analysed. Thirty-four were partially resistant to previous therapy with first-gen SSAs and the remaining 7 patients were completely resistant to first-gen SSA. At six months of treatments with Pasireotide Lar, 29 patients (70.7% of cases) reached the control of acromegaly. In this sub-analysis, we confirmed that Pasireotide Lar was preferred in patients that were partial resistant to first generation SSA (Supplementary Fig. 1). Moreover, we found that the complete resistance to SSA remained a risk factor for a poor response to Pasireotide Lar (OR: 6.8, 95%IC: 3–15.2; *p* < 0.001), as showed in Supplementary Fig. 2.

### Logistic regression

**Fig. 5:**
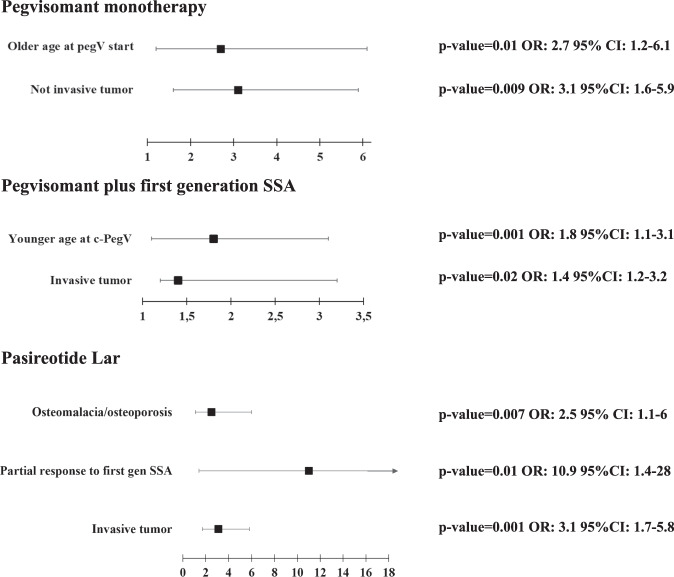
Forrest plot for the choice of second line therapies, logistic regression

The logistic regression data are summarized in Fig. 5 and confirmed that the main physicians’ determinants for choosing the treatment with Pegvisomant in monotherapy were the older patients age and the absence of tumor invasion. The choice of treatment with Pegvisomant in combination with first generation SSA was for the younger patient’s and the tumor invasion. The main physicians’ determinant for choosing the treatment with Pasireotide Lar was the diagnosis of osteomalacia or osteoporosis, the tumor invasion and the partial response to first generation SSA.

## Discussion

In this study, we retrospectively collected and analysed the clinical data of a cohort of acromegaly patients treated with second line therapies such as Pasireotide Lar and Pegvisomant in monotherapy or in combination with first-generation SSA, in a real-life experience that involved four hospital-based endocrinology centres in Rome, Italy. The patients that entered this study were all resistant to first generation SSA, having failed to reach the acromegaly control after at least 6 months of the maximum tolerated dose of treatment.

Among this cohort, thirty-three patients (33%) were treated with m-Pegvisomant in monotherapy, 36 (36%) with Pegvisomant plus first gen-SSAs and 31 patients with Pasireotide Lar (31%).

This study showed for the first time that the partial response achieved by the treatment with first gen-SSA may oriented physicians into choosing Pasireotide Lar as a second line treatment. In fact, we found that 94% of patients treated with Pasireotide Lar were considered partially responsive to first gen-SSA. Recently, Muhammad et al. [9] enrolled 52 acromegaly patients that were considered partially resistant to first-gen SSA and proved that the reduction of IGF-I correlated positively with the expression of the subtype 2 of the somatostatin receptor (SSTR2). In actual case series, we analysed the expression the SSTRs in 27 cases, according to the multicenter and retrospective design of this study, confirming our previous report on the prognostic role of the SSTR5 expression rather than of SSTR2A expression in predicting the response to Pasireotide Lar [10]. Data on the pharmacodynamic of Pasireotide Lar may justify our findings. In fact, it is well known that Pasireotide Lar binds the SSTR2A and SSTR5 (with a high affinity for the SSTR5) [11, 12]. We may hypothesize that the simultaneous interaction of Pasireotide Lar with the SSTR2A and SSTR5 should promote the normalization of GH and IGF-I, by the activation of the intracellular pathway of both these receptors. In addition, in this cohort, we found that all patients who reached the acromegaly control by Pasireotide Lar had been partially responsive to first-gen SSAs. At the same time, none case that was considered resistant to the treatment with first gen-SSA reached the biochemical control of acromegaly by Pasireotide Lar. In fact, we found that patients considered resistant to first gen-SSA had a 1.5-fold increased risk for a poor response to treatment with Pasireotide Lar.

As described in a previous multicenter cohort of acromegaly patients resistant to first gen-SSA [13], the actual study confirmed in a larger study population, the hypothesis that Peg-V plus first gen-SSA was more likely to be prescribed in patients with clinical/biochemical evidence of more severe/aggressive disease, as compared to those observed in patients treated by Pegvisomant in monotherapy. In this cohort we found that physicians prescribed Pegvisomant more frequently in monotherapy in older patients and in those with non-invasive tumors. Pegvisomant in combination with first gen-SSA was prescribed in younger patients, and those with invasive tumors, and Pasireotide Lar in cases with a partial response to first-gen SSA and in those with invasive tumors. The tendency to prescribe combination treatments with first gen-SSA and PEG-V in more severe and aggressive disease was described in our previous multi-center experience, despite data in Literature are not univocal [14–20].

In this study, we reported active acromegaly in 32 patients at the last follow-up: 11 patients were on treatment with m-Peg, 13 with c-Peg-V and 8 with Pasireotide Lar. We found that patients with active acromegaly during c-Peg-V therapy carried significantly higher IGF-I x ULN levels at the start of second line therapies (IGF-I x ULN: 3.4; IQR: 2.3), with respect to those treated with m-Peg-V (IGF-I x ULN: 2.3; IQR: 1.2, *p* = 0.004) and with Pasireotide Lar (IGF-I x ULN: 2; IQR: 0.7, *p* = 0.04). Also, these results supported and underlined the theory that patients treated with c-Peg-V were affected by a more aggressive/severe disease. This result confirmed our previous experience that an IGF-I superior to 3 x ULN may negatively predict the treatment outcome of Peg-V therapy [21] and required other treatment, such as combination treatment with Peg-V and Pasireotide Lar, radiosurgery or temozolomide [22, 23].

Despite our study was not being designed to provide data of efficacy of second line therapies, we found that at the follow-up last visit, the control of acromegaly was reached in 22 patients treated with m-Peg-V (66.7%), in 22 patients treated with c-Peg-V (63.9%) and in 25 patients treated with Pasireotide Lar (74.2%). These data are superimposable to those observed in previous series, as the reaching of acromegaly control is reported from 64% to 97% of patients treated with Peg-V in long term follow-up [24–35]. This magnitude in the percentage of patients who reached the control of acromegaly by second line therapies may be justified by the different study design (such as clinical trials, randomized or real life studies) and from the dosage of therapies. Data of efficacy on second line therapies are challenging to compare among the different series that may reflect the variability in severity disease. Our data of efficacy of Peg-V are similar to those reported by Van der Lely et al. [15], ranging around the 58% of treated patients and suggesting the need for a progressive increase of Peg-V dosage, to maintain the biochemical control of acromegaly. In fact, it was widely described that the rate of normalization of IGF-I increased during the treatment in parallel to the upper titration of therapeutic Peg-V dosage [8, 36]. The final PEG-V doses in that study were far lower than those recorded in our population, reflecting once again the severity of the disease in our patients. In fact, in our study we found a positive correlation between the Peg-V dosage (required for reaching the acromegaly control) and the levels IGF-I x ULN collected before starting the Peg-V treatment.

In this study, we conducted a sub-analysis to evaluate the efficacy of Pasireotide Lar at 6 months of treatment, in order to rule out a possible bias due to the evaluation of efficacy of Pasireotide Lar at 12 months of treatments, that may over-estimate the control rate. We found that the efficacy of Pasireotide Lar at 6 consecutive months of treatment was around 70% and resulted superimposable to the rate of control acromegaly observed in this study in patients treated for 12 consecutive months with Pasireotide Lar (74.2%). In addition, a 6 months treatment sub-analysis confirmed that the complete resistance to first gen-SSAs was as a risk factor for the poor response to Pasireotide Lar therapy.

In this study the regrowth of tumor residual occurred in one patient on treatment with m-Peg-V, one patient on treatment with c-Peg-V and one patient on treatment with Pasireotide Lar, the maintenance of first gen-SSA during treatment with Peg-V or the prescription of second generation SSA seem to be preferred in patients with tumor concern. This therapeutic approach is similar to those described in French Acrostudy, where the medical rationale for continuing SSAs rather than switching to PEG monotherapy was the tumour suprasellar extension and the reaching of tumour shrinkage during SSA [37]. However, recent data indicate that the fear of tumor re-growth during PEGV monotherapy is unfounded [38, 39] and that the regrowth of residual disease may be due to the biology of the neoplasm per-se, rather than to the effect of medical treatments [13]. Our data are in line with the hypothesis that tumor regrowth may be considered independent from the treatment choice, as this event occurred in one patient for each treatment. Moreover, in our patients’ cohort, we found that the frequency of diabetes mellitus at the choice of second-line therapy was similar among the three treatment groups, as 10 patients treated with m-Peg-V (25%), 11 patients treated with c-PegV (30.5%) and 11 patients treated with Pasireotide Lar (35.4%) were affected by diabetes mellitus. These data support the hypothesis that in centers devoted to the management of pituitary diseases the choice of second-line therapies may be guided by biochemical markers (as GH and IGF-I levels) and by the tumor mass features (such as invasiveness and residual disease) rather than by the occurrence of systemic complications of acromegaly, such as diabetes mellitus. Interestingly, in this study we found that patients affected by osteoporosis and/or osteomalacia were more likely be treated with Pasireotide Lar. Currently, in the absence of clear indications for the choice of second line therapies from the society guidelines, our data represent a real-life clinical experience [2–6].

The main limitation of this study is its retrospective design and the absence of unique treatment protocol among the different endocrine centers that enrolled patients for this study. These limitations did not allow us to provide data on the efficacy of second-line therapies, but allowed us to describe a real-life clinical experience. Moreover, according to the pathology protocol in the different centers, our study can’t provide complete data on the biology of GH secreting adenomas and on biomarkers (such as Ki67 Li, cytokeratin granulation pattern and the expression of somatostatin receptors) that may orient the choice of second-line treatments. At least, given the size and nature of our sample, it is difficult to tell whether and to what extent our observations on prescribing practices are indicative of practices in other hospitals in Italy or other countries. As for consequence, our results should be confirmed and validated in prospective studies, with larger cohort of patients, in particular with regard of the negative prognostic role of the resistance to first gen-SSA in predicting the poor outcome of Pasireotide Lar therapy, that we found in 7 patients treated for at least for 6 consecutive months with Pasireotide Lar and in 3 patients treated for 12 consecutive months.

Our results confirmed that Pasireotide Lar, Pegvisomant in monotherapy or in combination with SSA are safe and effective in reaching the control of acromegaly and underline that in SSA-resistant GH secreting pituitary adenomas, c-Peg-V and Pasireotide-Lar are choose for the treatment of invasive tumors. The partial response to first gen-SSA seem to be the main determinants that guides the choice of Pasireotide Lar.

## Supplementary Information

Figure1Supplementary

Figure2Supplementary

Supplementary Information

## Data Availability

Restrictions apply to the availability of some or all data generated or analyzed during this study to preserve patient confidentiality or because they were used under license. The corresponding author will on request detail the restrictions and any conditions under which access to some data may be provided. The data that support the findings of this study are available from the corresponding author upon reasonable request.
