# Supplementary figures and images for: Partial response to first generation SSA guides the choice and predict the outcome of second line therapy in acromegaly
Source: Endocrine. 2022 Aug 20;78(2):343–53. doi: 10.1007/s12020-022-03158-w (PMC9584996; doi:10.1007/s12020-022-03158-w)

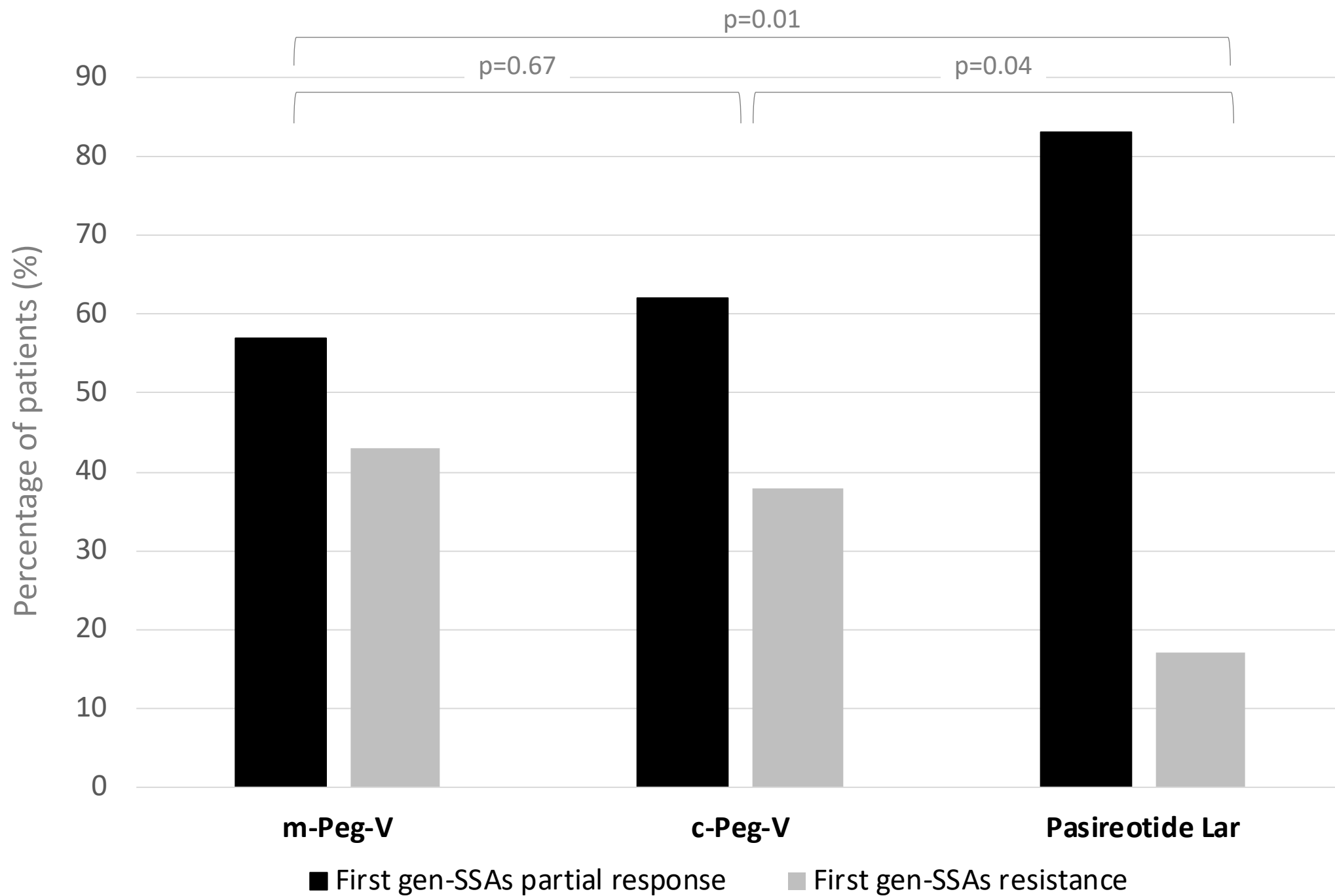

Supplement: Supplementary file 1 — Figure1Supplementary [file 12020_2022_3158_MOESM1_ESM.pdf]

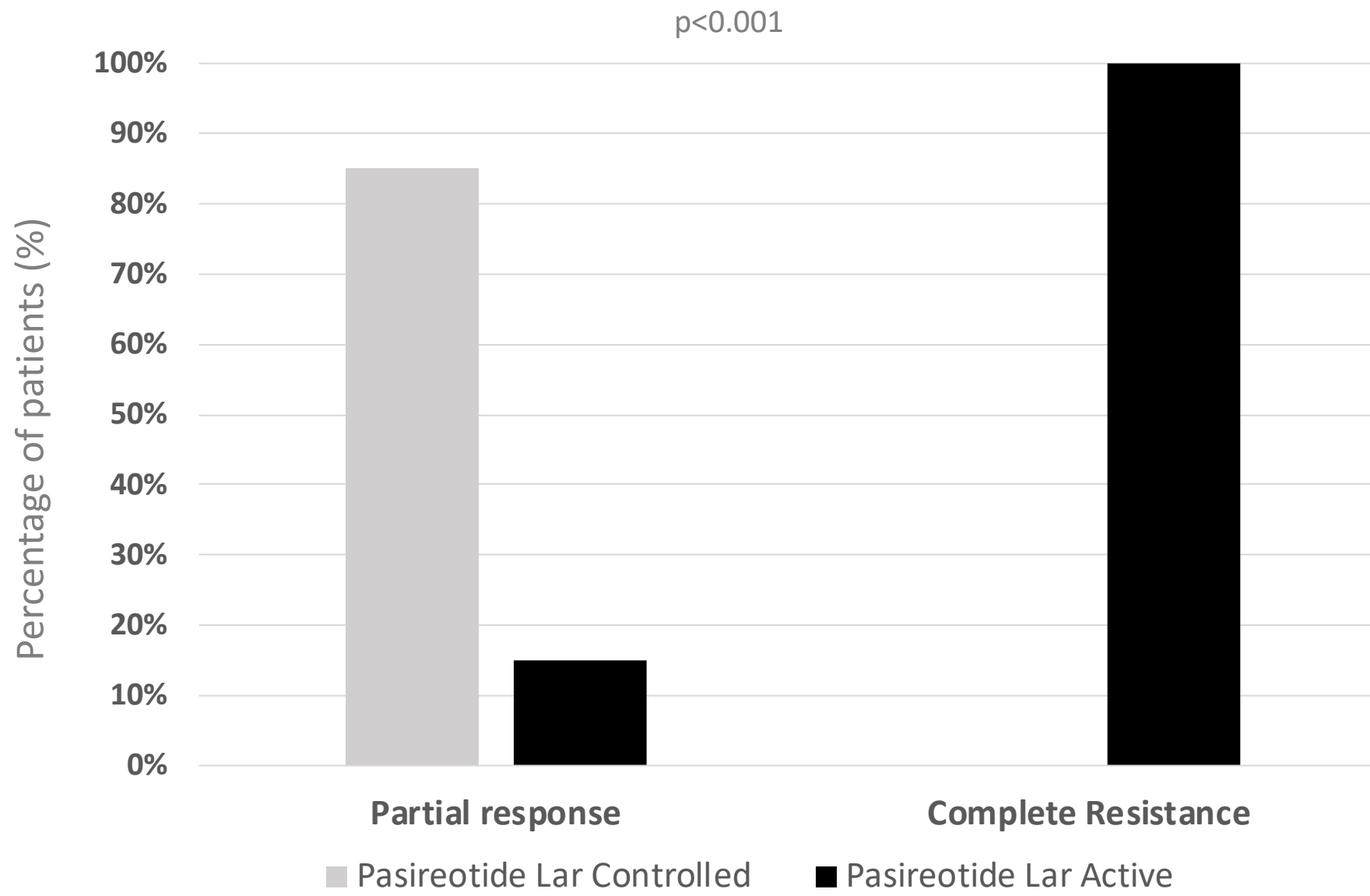

Supplement: Supplementary file 2 — Figure2Supplementary [file 12020_2022_3158_MOESM2_ESM.pdf]
