# Supplementary material for: Partial response to first generation SSA guides the choice and predict the outcome of second line therapy in acromegaly
Source: Endocrine. 2022 Aug 20;78(2):343–53. doi: 10.1007/s12020-022-03158-w (PMC9584996; doi:10.1007/s12020-022-03158-w)
Supplement: Supplementary file 3 — Supplementary Information [file 12020_2022_3158_MOESM3_ESM.docx]

Supplementary figure 1: Histogram representing the rate of partial control and resistance of first generation SSA according to the choice of different second line treatments (m-Peg-V, c-Peg-V and Pasireotide Lar), taking in account data of the sub-analysis for cases treated with Pasireotide Lar for at least 6 months. Univariate analysis.

Supplementary figure 2: Histogram representing the efficacy of Pasireotide Lar at 6 months of treatment, according to the response to first-gen SSA. Univariate analysis.
